# Inflammatory indexes in preoperative blood routine to predict early recurrence of hepatocellular carcinoma after curative hepatectomy

**DOI:** 10.1186/s12893-021-01180-9

**Published:** 2021-04-01

**Authors:** YiFeng Wu, ChaoYong Tu, ChuXiao Shao

**Affiliations:** 1 Department of Hepatobiliary and Pancreatic Surgery, The First Affiliated Hospital of Wenzhou Medical University, Nanbaixiang, Ouhai District, Wenzhou, Zhejiang People’s Republic of China; 2 Department of Hepatobiliary and Pancreatic Surgery, The Fifth Affiliated Hospital of Wenzhou Medical University, Lishui Municipal Central Hospital, 289 Kuocang Road, Liandu District, Lishui, Zhejiang People’s Republic of China

**Keywords:** Hepatocellular carcinoma, Inflammation, Recurrence, Hepatectomy, Prognosis

## Abstract

**Background:**

The inflammation indexes in blood routine play an essential role in evaluating the prognosis of patients with hepatocellular carcinoma, but the effect on early recurrence has not been clarified. The study aimed to investigate the risk factors of early recurrence (within 2 years) and recurrence-free survival after curative hepatectomy and explore the role of inflammatory indexes in predicting early recurrence.

**Methods:**

The baseline data of 161 patients with hepatocellular carcinoma were analyzed retrospectively. The optimal cut-off value of the inflammatory index was determined according to the Youden index. Its predictive performance was compared by the area under the receiver operating characteristic curve. Logistic and Cox regression analyses were used to determine the risk factors of early recurrence and recurrence-free survival.

**Results:**

The area under the curve of monocyte to lymphocyte ratio (MLR) for predicting early recurrence was 0.700, which was better than systemic inflammatory response index (SIRI), neutrophil to lymphocyte ratio (NLR), platelet to lymphocyte ratio (PLR) and systemic immune-inflammatory index (SII). MLR, tumour size, tumour differentiation and BCLC stage are all risk factors for early recurrence and recurrence-free survival of HCC. Combining the above four risk factors to construct a joint index, the area under the curve for predicting early recurrence was 0.829, which was better than single MLR, tumour size, tumour differentiation and BCLC stage. Furthermore, with the increase of risk factors, the recurrence-free survival of patients is worse.

**Conclusion:**

The combination of MLR and clinical risk factors is helpful for clinicians to identify high-risk patients with early recurrence and carry out active postoperative adjuvant therapy to improve the prognosis of patients.

## Background

Among the primary malignant tumours, liver cancer is the sixth most common, and the cancer fatality rate is the fourth. Among them, 75–85% of the pathological types are hepatocellular carcinoma (HCC) [1]. The new incidence of HCC in China accounts for about 50% of the world’s, and most HCC patients are complicated with hepatitis B [2]. Hepatectomy is one of the main treatments for HCC patients to achieve long-term survival. Even so, the postoperative cumulative recurrence rate within 5 years is about 70% [3], which has become the main cause of cancer-related death. According to the postoperative recurrence mechanism in patients with HCC, it can be divided into the early recurrence of intrahepatic metastasis and late recurrence of multicenter. Among them, early recurrence is more aggressive and the prognosis is worse [4, 5].

Hepatitis virus infection [6, 7], nonalcoholic fatty liver disease [8], alcohol [9], aflatoxin B1 [10] and other factors increase the risk of HCC. Although the mechanism of inducing tumourigenesis is different, inflammation is a common key link [11]. As an inflammatory-driven tumour, the occurrence, proliferation and metastasis of HCC are closely related to the inflammatory environment [12]. Therefore, the establishment of inflammatory indexes based on inflammatory cells in peripheral blood can reflect the relationship between tumour inflammatory microenvironment and the prognosis of HCC patients. At present, more and more evidence supports that systemic immune-inflammation index (SII) [13], neutrophil to lymphocyte ratio (NLR) [14], platelet to lymphocyte ratio (PLR) [15], monocyte to lymphocyte ratio (MLR) [16] and systemic inflammatory response index (SIRI) [17] are essential factors in predicting the survival of patients with HCC. However, there are still few reports about the role of the above inflammatory markers in predicting the early recurrence of HCC. Therefore, the purpose of our study is to explore the value of inflammatory indexes in blood routine in predicting early recurrence in patients with HCC and contrast their predictive performance.

## Materials and methods

**Fig. 1:**
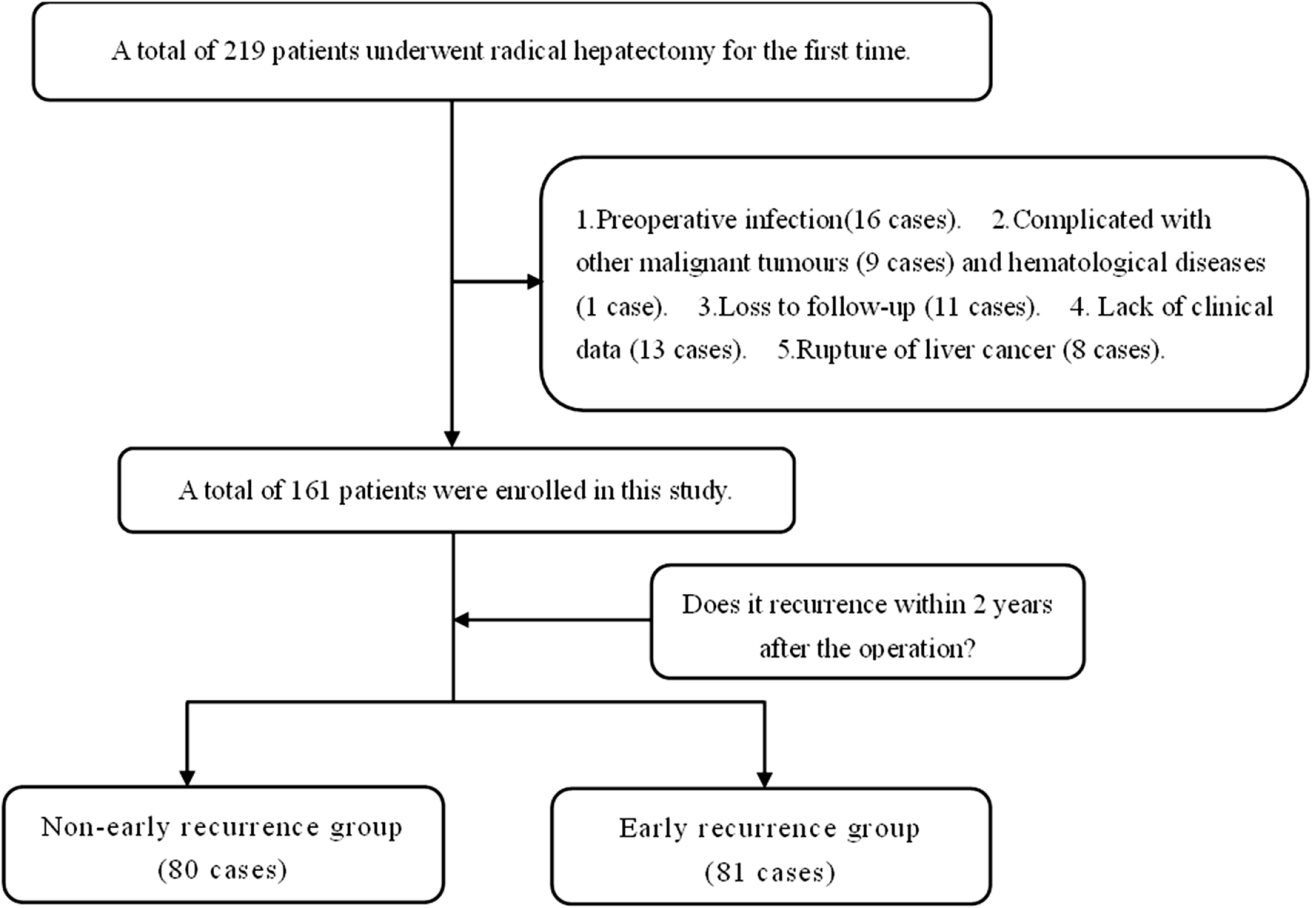
Flow chart for screening patients

The baseline data of patients undergoing curative hepatectomy in Lishui Municipal Central Hospital from January 2011 to May 2018 were collected and analyzed retrospectively. Inclusion criteria: (1) Postoperative pathology confirmed HCC; (2) Liver reserve function was Child–Pugh A or B grade; (3) Radical hepatectomy for the first time; (4) No distant metastasis was found in preoperative imaging examination and intraoperative metastasis. Exclusion criteria: (1) Lack of clinical data; (2) Suffer from infection or systemic inflammatory disease before the operation; (3) Complicated with other malignant tumours, immune or hematological diseases; (4) No regular follow-up or less than 2 years follow-up; (5) Preoperative treatment with immunosuppressants; (6) Rupture of liver cancer. Patients with HCC were closely followed up after the operation, and a total of 161 patients were enrolled in this study by May 2020 (Fig. 1). This study was in line with the Helsinki Declaration, it was reviewed and approved by the Ethics Committee of Lishui Municipal Central Hospital (Approval No. 2020-249).

### Follow-up

In the first 2 years after the operation, the patients were followed up every 3 months to the outpatient clinic. After 2 years, they were followed up every 6 months in the outpatient clinic until they relapsed or lost follow-up. The examination items included physical examination, serum alpha-fetoprotein test, and an abdominal imaging scan during the follow-up period. Besides, a chest computed tomography scan should be performed at least once a year. When abdominal ultrasound suspected recurrence, abdominal contrast-enhanced computed tomography scan or magnetic resonance imaging should be performed to confirm the diagnosis, while chest computed tomography enhanced scan should be performed to rule out pulmonary metastasis or positron emission tomography to rule out other site metastasis.

### Statistical analysis

The MedCalc11.0 version was used to draw the receiver operating characteristic curve. The optimal cut-off values of MLR, SIRI, NLR, PLR and SII were selected according to the Youden index. Meanwhile, the prediction performance is evaluated by comparing the area under the curve. The SPSS 22.0 version statistically analyzed the baseline data. The Pearson Chi-square test analyzed the counting data and logistic multivariate analysis model was used to obtain the independent risk factors of early recurrence after radical resection of HCC. Cox proportional hazards model was used to analyze the prognostic factors of recurrence-free survival (RFS). According to the number of risk factors, patients were divided into three groups by X-Tile software: low-risk group, medium-risk group and high-risk group. The survival curve was drawn by GraphPadPrism software, and the Log-Rank test compared the survival rate between groups. *P* < 0.05 considered that the difference was statistically significant.

### Data processing

Blood routine and blood biochemical results were collected within 1 week before the operation and calculated the inflammation index according to the following formula. SII = platelet × neutrophil/lymphocyte [13], NLR = neutrophil/lymphocyte [14], PLR = platelet/lymphocyte [15], MLR = monocyte/lymphocyte [16], SIRI = monocyte × neutrophil/lymphocyte [17], albumin–bilirubin grade (ALBI) = 0.66 × log10 [bilirubin (μmol/L)] − 0.085 × [albumin (g/L)] [18].

## Results

### Baseline data

A total of 161 patients were eligible for inclusion, including 141 males (87.6%) and 20 females (12.4%), with an average age of 56.24 ± 11.44 years. According to the definition of Child–Pugh grade, there were 157 patients with grade A and 4 patients with grade B. The average tumour size was 4.76 ± 3.15 cm. Since 21 patients had undergone transcatheter arterial chemoembolization and 3 patients had received radiofrequency ablation combined with transcatheter arterial chemoembolization before the operation, these patients need at least 1 month to consider surgical treatment. 149 patients (92.55%) were complicated with hepatitis B before the operation, and no hepatitis C patients were found. By the end of follow-up, a total of 96 patients (59.63%) relapsed, of which the 1 year, 2 years, 3 years and 5-year cumulative recurrence rates were 29.81%, 50.31%, 53.42%, 58.39%, respectively, and the median recurrence-free survival time was 23.9 months.

### The cut-off value and area under the curve of the inflammation index

**Fig. 2:**
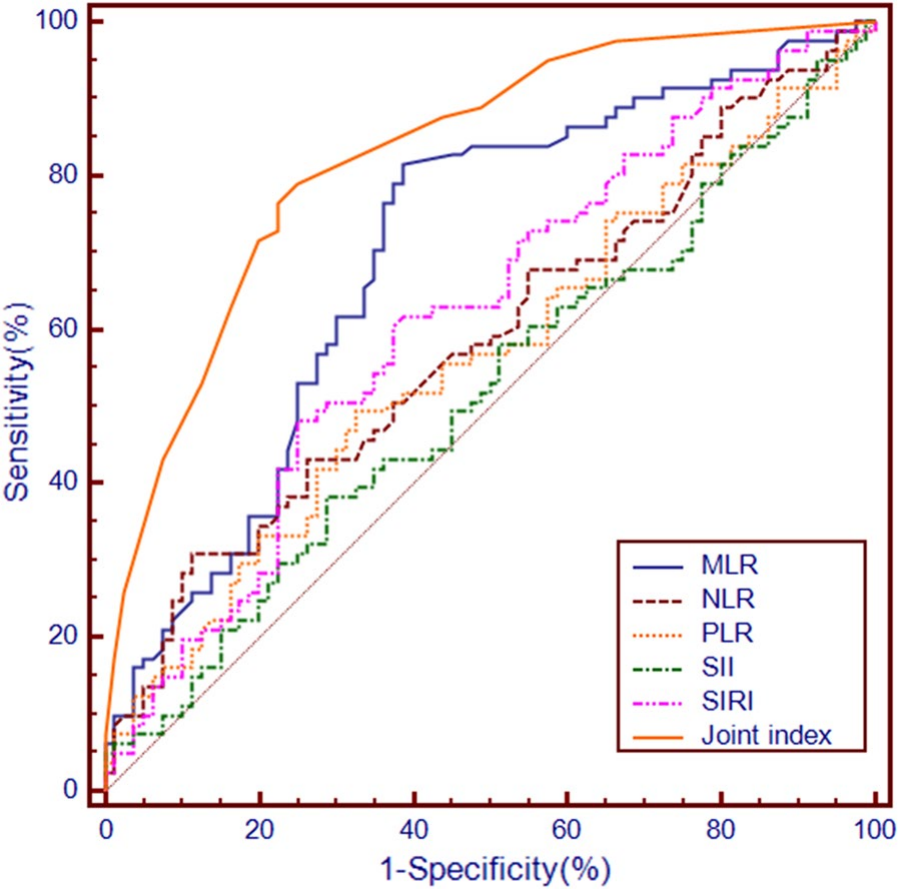
Comparison of the inflammatory index and joint index in predicting early recurrence

The receiver operating characteristic curves were drawn according to the preoperative MLR, SIRI, NLR, PLR and SII. The results showed that MLR = 0.25, SIRI = 1.03, NLR = 2.74, PLR = 88 and SII = 330 were the optimal cut-off values (Table 1). According to the cut-off value of the inflammation index, the patients who were less than or equal to the cut-off value were divided into the low group, and those who were greater than the cut-off value were divided into the high group. The area under the curve (AUC) of MLR, SIRI, NLR, PLR and SII were 0.700, 0.618, 0.587, 0.564 and 0.520, respectively. Based on the optimal cut-off value, the sensitivity of MLR, SIRI, NLR, PLR and SII was 81.5%, 48.1%, 30.9%, 49.4% and 38.3%, respectively, and the specificity was 61.2%, 75.0%, 88.7%, 67.5% and 71.2%, respectively. By comparing AUC (Fig. 2), it was found that MLR was superior to SIRI, NLR, PLR and SII in predicting early recurrence in patients with HCC.

**Table 1:** The cut-off value and area under the curve of the inflammation index

Factors	Cut-off value	AUC	Sensitivity (%)	Specificity (%)	Yoden index	95% CI of AUC
MLR	0.25	0.700	81.5	61.2	0.427	0.623–0.770
PLR	88	0.564	49.4	67.5	0.169	0.484–0.642
SII	330	0.520	38.3	71.2	0.095	0.440–0.599
NLR	2.74	0.587	30.9	88.7	0.196	0.507–0.664
SIRI	1.03	0.618	48.1	75.0	0.231	0.539–0.694

*AUC* area under curve, *CI* confidence interval, *MLR* monocyte to lymphocyte ratio, *PLR* platelet-to-lymphocyte ratio, *SII* systemic immune-inflammation index, *NLR* neutrophil to lymphocyte ratio, *SIRI* systemic inflammatory response index

### Univariate analysis of early postoperative recurrence

According to the recurrence within 2 years after the operation, the patients were divided into early recurrence group (81 cases) and non-early recurrence group (80 cases). The early recurrence group and non-early recurrence group’s clinicopathological characteristics were compared by univariate analysis of 22 factors (Table 2). The study found tumour size (*P* < 0.001), tumour number (*P* = 0.007), vascular invasion (*P* < 0.001), degree of differentiation (*P* < 0.001), MLR (*P* < 0.001), NLR (*P* = 0.001), PLR (*P* = 0.029), SIRI (*P* = 0.002), preoperative AFP (*P* = 0.023) and BCLC B–C stage (*P* < 0.001) were related to the early recurrence of HCC patients.

**Table 2:** Baseline clinicopathological characteristics of patients in early recurrence group and non-early recurrence group

Factors	Non-early recurrence group	Early recurrence group	X^2^	*P* value
	n = 80	n = 81		
Sex				
Male	68	73	0.971	0.324
Female	12	8		
Age (years)				
≤ 55	39	42	0.155	0.694
> 55	41	39		
AST (U/L)				
≤ 40	60	58	0.237	0.626
> 40	20	23		
ALT (U/L)				
≤ 40	49	51	0.050	0.823
> 40	31	30		
Total bilirubin (umol/L)				
< 17.1	60	60	0.018	0.893
≥ 17.1	20	21		
Albumin (g/L)				
< 35	7	15	3.256	0.071
≥ 35	73	66		
Tumour size (cm)				
≤ 5	63	40	15.061	< 0.001
> 5	17	41		
Tumour number				
Solitary	73	61	7.328	0.007
Multiple	7	20		
HBV infection				
No	6	6	0.001	0.982
Yes	74	75		
Liver cirrhosis				
No	21	20	0.052	0.820
Yes	59	61		
Vascular invasion				
No	75	57	14.897	< 0.001
Yes	5	24		
Differentiation				
Moderate and well	61	37	15.793	< 0.001
Poor	19	44		
Blood transfusion				
No	48	46	0.171	0.680
Yes	32	35		
Hepatic hilar occlusion				
No	41	40	0.056	0.813
Yes	39	41		
SII				
≤ 330	57	51	1.252	0.263
> 330	23	30		
MLR				
≤ 0.25	49	16	28.790	< 0.001
> 0.25	31	65		
NLR				
≤ 2.74	71	55	10.283	0.001
> 2.74	9	26		
PLR				
≤ 88	54	41	4.743	0.029
> 88	26	40		
SIRI				
≤ 1.03	60	42	9.289	0.002
> 1.03	20	39		
AFP (ng/mL)				
< 400	65	53	5.145	0.023
≥ 400	15	28		
ALBI grade				
1	47	40	1.422	0.233
2	33	41		
BCLC stage				
0–A	68	47	14.351	< 0.001
B–C	12	34		

*AST* aspartate aminotransferase, *ALT* alanine aminotransferase, *HBV* hepatitis B virus, *AFP* alpha fetoprotein, *ALBI* albumin–bilirubin, *BCLC* Barcelona Clinic Liver Cancer

### Multivariate analysis of early recurrence

The statistical significance factors in univariate analysis were included in the Logistic multivariate regression model (Table 3). The results showed that high MLR, tumour size > 5 cm, poor differentiation and BCLC stage were independent risk factors for early recurrence after radical resection of HCC (*P* < 0.05). The corresponding AUC was 0.700, 0.647, 0.653 and 0.635, respectively (Table 4).

**Table 3:** Logistic multivariate analysis of early recurrence

Factors	β coefficient	OR	95% CI	P value
Tumour size > 5 cm	1.036	2.819	1.189–6.679	0.019
Multiple tumours	0.688	1.989	0.589–6.714	0.268
Vascular invasion	0.931	2.537	0.719–8.955	0.148
Poor differentiation	1.104	3.017	1.257–7.242	0.013
High MLR	1.442	4.227	1.573–11.357	0.004
High NLR	0.655	1.926	0.620–5.980	0.257
High PLR	− 0.021	0.979	0.407–2.355	0.962
High SIRI	0.017	1.017	0.346–2.994	0.975
AFP ≥ 400	0.304	1.355	0.475–3.868	0.570
BCLC B–C stage	1.107	3.026	1.139–8.039	0.026

*OR* odds ratio

**Table 4:** Area under the curve of risk factors for early recurrence

Factors	AUC	Sensitivity (%)	Specificity (%)	Youden index	95% CI of AUC
Differentiation	0.653	54.3	76.2	0.305	0.574–0.726
MLR	0.700	81.5	61.2	0.427	0.623–0.770
Tumour size	0.647	50.6	78.7	0.293	0.568–0.720
BCLC stage	0.635	42.0	85.0	0.270	0.555–0.709
Joint index	0.829	76.5	77.5	0.540	0.761–0.883

### Univariate and multivariate analysis of recurrence-free survival

In univariate analysis (Table 5), the study found that tumour size (*P* < 0.001), tumour number (*P* < 0.001), vascular invasion (*P* < 0.001), degree of differentiation (*P* < 0.001), MLR (*P* < 0.001), NLR (*P* < 0.001), SIRI (*P* < 0.001), preoperative AFP (*P* = 0.035) and BCLC stage (*P* < 0.001) were factors affecting recurrence-free survival (RFS).

**Table 5:** Univariate and multivariate analysis of recurrence-free survival

Factors	Univariate analysis			Multivariate analysis		
	HR	95% CI	*P* value	HR	95% CI	*P* value
Sex (male/female)	1.436	0.722–2.855	0.302	–	–	–
Age (≤ 55/> 55 years)	0.882	0.591–1.317	0.540	–	–	–
AST (≤ 40/> 40 U/L)	1.316	0.854–2.027	0.213	–	–	–
ALT (≤ 40/> 40 U/L)	1.018	0.674–1.538	0.933	–	–	–
Total bilirubin (< 17.1/≥ 17.1 umol/L)	1.032	0.649–1.642	0.893	–	–	–
Albumin (> 35/≥ 35 g/L)	0.678	0.393–1.167	0.160	–	–	–
Tumour size (≤ 5/> 5 cm)	2.283	1.523–3.422	< 0.001	1.768	1.144–2.732	0.010
Tumour number (solitary/multiple)	2.421	1.495–3.919	< 0.001	2.289	1.295–4.047	0.004
HBV infection (no/yes)	0.904	0.438–1.865	0.785	–	–	–
Liver cirrhosis (no/yes)	1.095	0.694–1.728	0.696	–	–	–
Vascular invasion (no/yes)	2.924	1.845–4.635	< 0.001	1.908	1.162–3.132	0.011
Differentiation (moderate and well/poor)	2.490	1.662–3.732	< 0.001	2.411	1.521–3.824	< 0.001
Blood transfusion (no/yes)	1.184	0.790–1.773	0.413	–	–	–
Hepatic hilar occlusion (no/yes)	1.130	0.757–1.687	0.551	–	–	–
SII (≤ 330/ > 330)	1.382	0.911–2.098	0.129	–	–	–
MLR (≤ 0.25/> 0.25)	3.801	2.341–6.171	< 0.001	2.037	1.154–3.597	0.014
NLR (≤ 2.74/> 2.74)	2.513	1.599–3.950	< 0.001	2.070	1.212–3.537	0.008
PLR (≤ 88/> 88)	1.404	0.938–2.100	0.099	–	–	–
SIRI (≤ 1.03/> 1.03)	2.248	1.496–3.378	< 0.001	1.320	0.773–2.255	0.309
AFP (< 400/≥ 400 ng/mL)	1.587	1.034–2.437	0.035	0.914	0.557–1.499	0.721
ALBI grade (1/2)	1.180	0.787–1.768	0.423	–	–	–
BCLC stage (0–A/B–C)	2.467	1.628–3.740	< 0.001	1.969	1.216–3.187	0.006

*HR* hazard ratio

Significant factors in COX univariate analysis were included in COX multivariate regression model. The results showed that tumour size > 5 cm (HR 1.768, 95% CI [1.144–2.732]), multiple tumours (HR 2.289, 95% CI [1.295–4.047]), vascular invasion (HR 1.908, 95% CI [1.162–3.132]), poor differentiation (HR 2.411, 95% CI [1.521–3.824]), high MLR (HR 2.037, 95% CI [1.540–3.597]), high NLR (HR 2.070, 95% CI [1.212–3.537]) and BCLC B–C stage (HR 1.969, 95% CI [1.216–3.187]) were independent risk factors for RFS in HCC patients (*P* < 0.05).

### Recurrence-free survival curve of inflammatory markers in blood routine

**Fig. 3:**
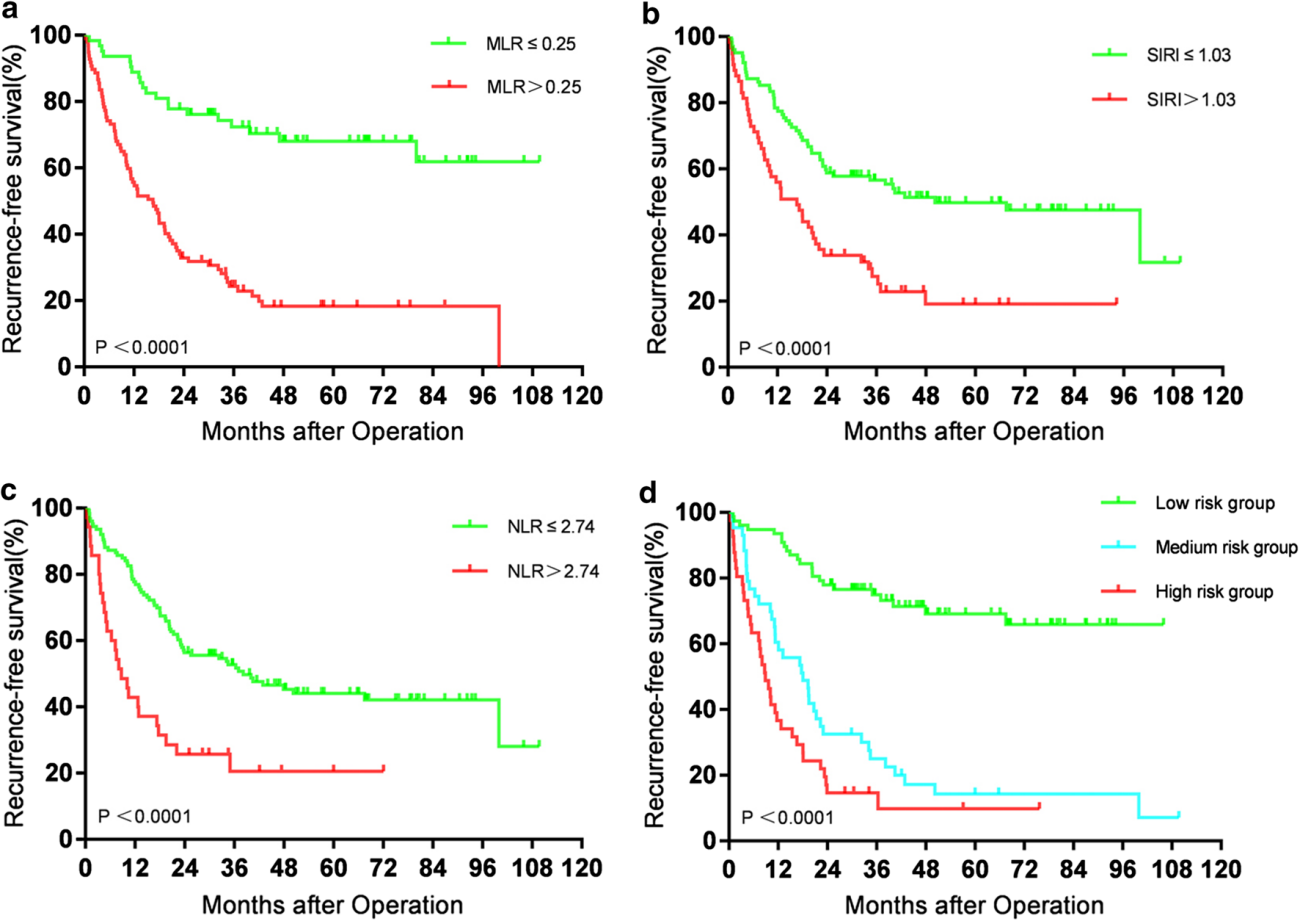
**a** Recurrence-free survival curves of patients with high MLR and low MLR. **b** Recurrence-free survival curve of patients with high SIRI and low SIRI. **c** Recurrence-free survival curve of patients with high NLR and low NLR. **d** Recurrence-free survival curves of low-risk, intermediate-risk and high-risk patients

The survival curves of the two groups were drawn by Kaplan–Meier. The results showed that the recurrence-free survival rate of the high MLR group, high SIRI group, and high NLR group were all significantly lower than that of the low MLR group, low SIRI group, and low NLR group (*P* < 0.0001) (Fig. 3a–c). The high SII and high PLR group was compared separately with the low SII and low PLR group. The high group’s recurrence-free survival rate was not significantly different from those in the low group (*P* = 0.127, *P* = 0.098).

### Combined risk factors to predict early recurrence and recurrence-free survival

The results showed that MLR, tumour size, differentiation and BCLC stage were independent risk factors for early recurrence and recurrence-free survival. The β coefficient is taken as the weight of the four risk factors to construct a joint index. Joint index = 1.036 * tumour size + 1.104 * differentiation + 1.107 * BCLC stage + 1.442 * MLR, MLR (low group MLR assignment is 0, high group MLR assignment is 1), tumour size (tumour size ≤ 5 cm assignment is 0, tumour size > 5 cm assignment is 1), differentiation degree (high-middle differentiation assignment is 0, low differentiation assignment is 1). The AUC, sensitivity and specificity of the joint index for predicting early recurrence were 0.829, 76.5% and 77.5%, respectively. The predictive performance was better than that of single MLR, tumour size differentiation, and BCLC stage (Table 4).

Similarly, in the recurrence-free survival, according to the four risk factors of MLR, tumour size, differentiation and BCLC stage, the patients were divided into the low-risk group when they had 0–1 risk factors, medium risk group when they had 2 risk factors and high-risk group when they had more than 3 risk factors. The results showed that there was a significant difference in recurrence-free survival rate between the high-risk group and middle-risk group (*P* < 0.049) and between the high-risk group and low-risk group (*P* < 0.0001). Similarly, there was a significant difference in the recurrence-free survival rate between the middle-risk and low-risk groups (*P* < 0.0001). The recurrence-free survival rate was the best in the low-risk group, the second in the middle-risk group, and the worst in the high-risk group (Fig. 3d).

## Discussion

Since tumour-associated inflammation has become one of the top ten characteristics of tumours [19], the value of inflammatory indexes in evaluating patients’ prognosis with HCC has been affirmed. However, there are few studies on the relationship between inflammatory indexes and early recurrence. At present, there is no consensus on time cut-off point of early recurrence, and the definition of early recurrence varies from 6 months to 2 years in most studies [5, 20, 21]. Recurrence accounted for more than 70% of HCC recurrence within 2 years after hepatectomy [22], and the follow-up of this study found that the peak of HCC recurrence was mainly within 2 years, and the cumulative recurrence rate of 2 years was 50.31%. Then the recurrence rate tended to be flat, so the cut-off point of early recurrence was set within 2 years in this study.

However, at present, it is generally accepted that early recurrence may initially originate from the occult micrometastasis of the tumour, which is usually related to the characteristics of the invasive tumour, such as multiple tumours, large tumour size, macrovascular and microvascular invasion, poorly differentiated tumour and satellite nodules [23–26]. In this study, multivariate analysis showed that multiple tumours and vascular invasion were not risk factors for early recurrence. It may be due to tumour necrosis caused by preoperative interventional therapy such as radiofrequency ablation and transarterial chemoembolization [27], affecting the postoperative pathological evaluation of the tumour. However, in the RFS of patients with HCC, the results confirmed that high MLR, high NLR, vascular invasion, multiple tumours, tumour size > 5 cm, poor differentiation and BCLC B–C stage were independent risk factors.

There have been few reports about the predictive value of inflammatory markers in early recurrence in the past. This study found that the predictive value of MLR for early recurrence is better than NLR, SIRI, PLR, and SII. Meanwhile MLR is a risk factor for early recurrence and RFS, reflecting the enhancement of inflammatory response to promote tumour cell proliferation and metastasis and the weakening of immune response to induce tumour cell apoptosis and inhibit tumour cell invasion. Considering that a single MLR does not have an ideal predictive performance, this study combines inflammatory index with clinical factors to evaluate tumour invasiveness more comprehensively from different angles, which can improve the accuracy of predicting early recurrence and RFS of patients with liver cancer, identify high-risk patients, adopt active and effective preventive and therapeutic measures, and strengthen follow-up observation, to prolong the survival time of patients.

At present, the mechanism of inflammation and tumour is not precise. However, neutrophils, platelets and monocytes are representative inflammatory cells in blood routine, and some studies have proposed the relationship between neutrophil, platelet and tumour. In the initial stage of the tumour, tumour-associated macrophages formed by neutrophils and monocytes will release reactive oxygen species, destroying gene stability and causing gene mutation, leading to tumorigenesis [28, 29]. Subsequently, in the stage of tumour proliferation and metastasis, neutrophils, platelets and tumour-associated macrophages secrete a variety of proteins, enzymes and factors, which promote tumour angiogenesis, induce tumour cell epithelial–mesenchymal transformation and participate in extracellular matrix remodeling. It not only provides favourable conditions for tumour growth but also enhances the migration and invasion ability of tumour cells [28–30]. Besides, platelets form a protective film on tumour cells’ surface to protect them from blood flow shear stress while escaping natural killer cells’ immunity [30] and the release of external neutrophil trapping network, which promotes tumour metastasis [29]. Finally, neutrophils and tumour-associated macrophages mediate the suppression of anti-tumour immunity by inhibiting the immune function of natural killer cells and T cells, resulting in malignant progression [28, 29].

In this study, an interesting phenomenon was that the predictive performance of inflammatory markers (PLR and SII) containing platelets was lower than that of SIRI, NLR and MLR. Because HCC patients often experience a chronic liver disease process in which the liver parenchyma is continuously destroyed and then replaced by fibrous tissue under the induction of the hepatitis virus, non-alcoholic fatty liver, alcohol and other causes. Thrombopoietin is an essential factor in regulating megakaryocyte maturation and platelet production [31]. Hepatocytes are the primary source of thrombopoietin production [32]. In the process of chronic liver disease, the number of functional hepatocytes decreases, thus reducing the secretion of thrombopoietin. In addition, cirrhosis and portal hypertension can cause hypersplenism, trapping platelets in the spleen [33] and destroys platelets through macrophages, resulting in thrombocytopenia in the peripheral blood [34]. Therefore, the platelet count can often reflect the severity of chronic liver disease and portal hypertension [35]. Moreover, some of the patients included in this study were complicated with hypersplenism, while others had undergone splenectomy. The above factors will affect the platelet level, thus interfere with the accuracy of PLR and SII in predicting early recurrence. It can be seen that compared with other inflammatory indicators, inflammatory indicators containing platelets, such as PLR and SII, are not effective and have more limitations in patients with HCC.

The study has some shortcomings because this is a single-centre retrospective cohort study, the sample size is small, it can not effectively control confounding factors, there will inevitably be deviations, affecting the quality of the study. Secondly, the aetiology and race of patients with HCC are diverse, which leads to the inconsistent selection of the optimal cut-off value of inflammatory indicators in different studies, which interferes with the results to a certain extent. Therefore, prospective verification is needed in large-scale multicenter studies.

## Conclusions

To sum up, as a simple, cheap and convenient inflammatory index, MLR can predict early recurrence and RFS in patients with HCC and will have a more excellent application prospect when combined with other clinical risk factors.

## Data Availability

The datasets generated and/or analyzed during the current study are not publicly available due to patient privacy and security of electronic medical information but are (anonymized) available from the corresponding author on reasonable request.

## Abbreviations

HCC: Hepatocellular carcinoma
MLR: Monocyte to lymphocyte ratio
SIRI: Systemic inflammatory response index
NLR: Neutrophil to lymphocyte ratio
PLR: Platelet to lymphocyte ratio
SII: Systemic immune-inflammatory index
RFS: Recurrence-free survival
AUC: Area under the curve
